# Investigating the “Fetal Side” in Recurrent Pregnancy Loss: Reliability of Cell-Free DNA Testing in Detecting Chromosomal Abnormalities of Miscarriage Tissue

**DOI:** 10.3390/jcm12123898

**Published:** 2023-06-07

**Authors:** Silvia D’Ippolito, Giuliana Longo, Daniela Orteschi, Andrea Busnelli, Nicoletta Di Simone, Eleonora Pulcinelli, Giorgia Schettini, Giovanni Scambia, Marcella Zollino

**Affiliations:** 1 Dipartimento della Salute della Donna, del Bambino e di Sanità Pubblica, Fondazione Policlinico Universitario A. Gemelli, Istituto di Ricovero e Cura a Carattere Scientifico (IRCCS), 00168 Rome, Italy; giovanni.scambia@policlinicogemelli.it; 2 BioRep Srl, Centro di Risorse Biologiche, Sapio Group, 20900 Milan, Italy; 3 Dipartimento Universitario Scienze della Vita e Sanità Pubblica, Sezione di Medicina Genomica, Università Cattolica del Sacro Cuore, 00168 Rome, Italy; 4 Department of Biomedical Sciences, Humanitas University, 20072 Milan, Italy; 5 IRCCS Humanitas Research Hospital, 20089 Rozzano, Italy; 6 Università Cattolica del Sacro Cuore, 00168 Rome, Italy; giorgiaschettini@gmail.com; 7 Dipartimento Universitario Scienze della Vita e Sanità Pubblica, Università Cattolica del Sacro Cuore, 00168 Rome, Italy; 8 Genetica Medica, Fondazione Policlinico Universitario A. Gemelli, Istituto di Ricovero e Cura a Carattere Scientifico (IRCCS), 00168 Rome, Italy

**Keywords:** recurrent pregnancy loss, spontaneous miscarriage, non-invasive prenatal testing, genetic testing

## Abstract

(1) Background: The aim of our study is to evaluate whether cell-free DNA testing can overlap the genetic testing of miscarriage tissue in women with early pregnancy loss (EPL) and length of recurrent pregnancy loss (RPL); (2) Methods: We conducted a prospective cohort study at the Pregnancy Loss Unit of the Fondazione Policlinico Universitario A. Gemelli (IRCCS), Rome, Italy between May 2021 and March 2022. We included women with EPL and length of RPL. Gestational age was >9 weeks + 2 days and <12 weeks + 0 days of gestation corresponding to a crown rump length measurement of >25 and <54 mm. Women underwent both dilation and curettage for the collection of miscarriage tissue and for blood sample collection. Chromosomal microarray analysis (CMA) on miscarriage tissues was performed by oligo-nucleotide- and single nucleotide polymorphisms (SNP)-based comparative genomic hybridization (CGH+SNP). Maternal blood samples were analyzed by Illumina VeriSeq non-invasive prenatal testing (NIPT) to evaluate the cell-free fetal DNA (cfDNA) and the corresponding fetal fraction and the presence of genetic abnormalities; (3) Results: CMA on miscarriage tissues revealed chromosome aneuploidies in 6/10 cases (60%), consisting of trisomy 21 (5 cases) and monosomy X (one case). cfDNA analysis was able to identify all cases of trisomy 21. It failed to detect monosomy X. A large 7p14.1p12.2 deletion concomitant to trisomy 21 was, in one case, detected by cfDNA analysis but it was not confirmed by CMA on miscarriage tissue. (4) Conclusions: cfDNA largely reproduces the chromosomal abnormalities underlying spontaneous miscarriages. However, diagnostic sensitivity of cfDNA analysis is lower with respect to the CMA of miscarriage tissues. In considering the limitations when obtaining biological samples from aborted fetuses suitable for CMA or standard chromosome analysis, cfDNA analysis is a useful, although not exhaustive, tool for the chromosome diagnosis of both early and recurrent pregnancy loss.

## 1. Introduction

Miscarriage, defined as pregnancy loss before 20 weeks of gestation, is a frequent obstetrical complication affecting up to 15–25% of all clinically diagnosed pregnancies [1]. In the general population, it has been estimated that about 50% of miscarriages occurring before 10 weeks of gestation are due to chromosomal abnormalities. Trisomies are the most frequently detected abnormalities, followed by triploidies, monosomy X, tetraploidies, and structural chromosome anomalies [1,2,3,4]. Chromosome abnormality also plays a relevant role in the pathophysiology of recurrent pregnancy loss (RPL) [5,6,7]. The probability of carrying a chromosome abnormality in couples with two or more miscarriages is significantly higher than in the general reproductive age population and varies between 3.2% and 5.8% [8]. Notably, the prevalence of abnormalities in women with two losses has been observed to be higher than that in women with three or more losses [5,6,7,9]. Not surprisingly, according to the recent European Society of Human Reproduction and Embryology (ESHRE) guideline on RPL, genetic analysis of miscarried tissue has the benefit of providing the patient with a reason for the pregnancy loss and may help to determine whether further investigations or treatments are required [10,11,12,13,14,15,16,17,18]. Identifying the cause of RPL is important, not only for its prognostic implications, but also for satisfying a couple’s desire to know the reason underlying their pregnancy loss. A survey constructed to assess the public perceptions of miscarriage reported that up to 75% of all respondents strongly wished to know the cause of their pregnancy loss, even if no intervention could have prevented it from occurring. In addition, 47% of them felt guilty, 41% reported feeling that they had done something wrong, 41% felt left on their own, and 28% felt ashamed [10]. Although studies dealing with the psychological benefit deriving from cytogenetic testing are still scant, this survey suggested that identifying a potential cause of the miscarriage may influence patients’ psychological responses to this unfortunate event [10]. Conventional karyotyping has been the most used genetic test for the genetic analysis of pregnancy tissue for over 30 years [19]. However, its several limitations (i.e., high rates of culture failure and bacterial and maternal cell contamination, high costs, waiting time for results, laboratory workload, etc.) have prompted the introduction of alternative techniques [3,10,11,20,21,22].

Currently, array comparative genomic hybridization (array-CGH) and single nucleotide polymorphism array (SNP-array) are considered the most reliable tests and are recommended by the most authoritative guidelines on this issue [3,11,23,24,25,26,27,28]. Nevertheless, chromosomal microarray analysis (CMA) also has some relevant weaknesses including the inability to detect balanced chromosomal rearrangements and low-level mosaicism. Notably, the array-CGH also does not reliably detect all polyploidies [3,23]. Finally, both the costs and the percentage of inconclusive results due to the maternal origin of the obtained specimens during uterine dilation and curettage, limit the large-scale use of CMA in pregnancy loss [29,30].

In recent years, prenatal screening of chromosomal abnormalities has undergone rapid development, with advances in molecular technology driving the change. Non-invasive prenatal testing (NIPT), based on sequencing of cell-free DNA (cfDNA) in maternal plasma, is a highly sensitive and effective screening test for chromosomal abnormalities [31]. The rapid evolution of the underlying technology, the absence of the risks of invasive methods and the favorable cost-effectiveness ratio, have favored the diffusion of this test not only in the private sector but also in the public sector. Indeed, some national health systems offer NIPT, particularly to pregnant women with a probability of a rare condition identified in the fetus, following the combined first-trimester screening [32]. Considering the recognized importance of obtaining information about the etiology of RPL, herein we hypothesize that cfDNA could be a valid test to identify chromosomal abnormalities of the product of conception in case of spontaneous abortion. In addition to the known advantages, NIPT, in this context, could provide information about the karyotype of the aborted embryo/fetus even in those cases in which it is usually more difficult to obtain, i.e., women who have miscarried outside of the hospital setting, women opting for medical treatment, etc. To the best of our knowledge, there is little evidence about the reliability of cfDNA in identifying the genetic causes underlying RPL. Against that background, the aim of the present pilot study was to investigate the overlapping diagnostic yield of cfDNA analysis and CMA of miscarriage tissues in women with RPL.

## 2. Materials and Methods

### 2.1. Patients

This study was performed between May 2021 and March 2022 at the Pregnancy Loss Unit, Dipartimento di Scienze della Salute della Donna, del Bambino e di Sanità Pubblica, Fondazione Policlinico Universitario A. Gemelli IRCCS, Università Cattolica del Sacro Cuore, Rome, Italy. The study population included women diagnosed with miscarriage (<12 weeks of gestation) with pregnancy tissue in situ clinically documented by ultrasonography and history of idiopathic RPL. Inclusion criteria were as follows: age >18 years; intrauterine singleton spontaneous pregnancy loss diagnosed according to international guidelines [15,33,34]; embryo crown rump length (CRL) measurement >24 and ≤54 mm equivalent to a gestational age between 9 weeks + 1 days and 12 weeks + 0 days; history of ≥3 previous spontaneous pregnancy losses; acceptance of dilation and curettage (D&C) procedure as treatment for miscarriage. Exclusion criteria were as follows: ultrasound diagnosis of blighted ovum (empty gestational sac); ultrasound finding suggestive of molar pregnancy; multiple uterine fibroids or single uterine fibroid of more than 4 cm in mean diameter; body mass index (BMI) > 25 Kg/m^2^; parental karyotype abnormalities; known current malignancy; blood transfusions or stem cell therapy or immunotherapy within the previous 3 months; previous organ transplantation; current psychiatric disorder requiring treatment; positive hepatitis B, hepatitis C, human immunodeficiency virus (HIV) status; SARS-CoV-2 coronavirus infection; inability to understand or sign the informed consent.

All women gave their informed consent to use, anonymously, their data for research purposes, and the protocol was approved by the ethics committee of the Fondazione Policlinico Universitario A. Gemelli IRCCS, Università Cattolica del Sacro Cuore in Rome, Italy (protocol ID 3868).

All women underwent an obstetric ultrasound, performed by an operator with at least 3 years of experience. A Samsung machine (Samsung) with a 9 MHz frequency probe was used. Crown rump length (CRL) measurement was obtained by a two-dimensional ultrasound (2DUS) on the midsagittal plane according to the International Society Ultrasound in Obstetrics and Gynecology (ISUOG) Practice Guidelines [12].

Genetic counselling was offered to all study participants before undergoing the genetic test. In particular, participants were informed of the possible (i) unsuccessful outcome of the analysis due to inadequate fetal fraction (FF, expressed as percentage); (ii) presence of a result with quantitative chromosome changes of uncertain significance; (iii) presence of medically actionable incidental findings. Women were also informed that cfDNA analysis would have been performed only if the collected products of conception (POC) were adequate for the CMA.

### 2.2. Biological Samples Collection

Participating women underwent dilation and curettage (D&C) for miscarriage tissue collection. Spontaneous miscarriage tissue samples were gently collected during the D&C procedure and no suction was performed. The samples were collected in a sterile tube and labelled with the patient’s name and/or identification number and the collection date. Collected samples were stored at 4 °C and sent to the Medical Genetics Service, Fondazione Policlinico Universitario A. Gemelli IRCCS (Rome) within 3 days of the day of collection. Blood samples were collected in a sterile tube (10 mL) on the same day of the programmed D&C procedure, before any infusion of anesthetic drugs or fluids. The collected tubes were labelled with the patient’s name and/or identification number and the collection date. The aliquots for the analysis of cfDNA were sent to Biorep Gemelli s.r.l. Laboratory (Milan) within two days of the day of collection.

### 2.3. Spontaneous Miscarriage Tissue Processing: Pathological Examination and Chromosomal Microarray Analysis

The macroscopic evaluation of the miscarriage tissue was performed in the Department of Pathology of the Fondazione Policlinico Universitario A. Gemelli IRCCS, Università Cattolica del Sacro Cuore in Rome, Italy. The fresh specimens were evaluated by one pathologist to isolate the embryo/fetal component in the miscarriage tissue from the whole fresh miscarriage specimen. The criteria for a macroscopic identification of the embryo/fetal component required: a consistent pattern, a color that tends toward a translucent grey and the embryo/fetal shape. The isolated embryo/fetal component was collected in a length of tube. The remaining tissue underwent a routine histological examination. The genetic examination was performed through an oligo-nucleotide-based and SNP (single nucleotide polymorphisms)-based comparative genomic hybridization (CGH+SNP) microarray analysis (CGH+SNP ISCA Microarray kit 4 × 180K, 110,712 oligonucleotide and 59,647 SNP probes, alignment on NCBI 37 (UCSC hg19) (Agilent Technologies, Santa Clara, CA, USA).

The amount of fetal tissue required for CMA was about 5 ± 10 mg. The isolated miscarried tissue underwent gender assessment by looking at the presence or absence of sex-determining region Y (SRY). Subsequently oligo-CGH+SNP microarray analysis was performed. When the embryo/fetal component was not easily identifiable, the following actions were taken: (1) a further pathological evaluation was performed in order to isolate the embryo/fetal component from the whole miscarriage tissue; the remaining tissue underwent the histological examination; (2) a microsatellite segregation analysis (MSA) on both maternal and likely embryo/fetal DNA of the isolated component was performed; (3) oligo-CGH+SNP microarray analysis was performed on those samples that were shown not to be contaminated. For histological analysis, the miscarriage specimens were fixed in 10% buffered formaldehyde for 20 ± 24 h, embedded in paraffin and 5-micron-thick microtomic sections were stained with hematoxylin-eosin.

### 2.4. Cell-Free DNA (cfDNA) Analysis

We only tested blood samples where the collection of POC was adequate for the CMA. Analysis of circulating cfDNA in maternal plasma was undertaken by a whole genome sequencing (WGS) from a single tube of maternal blood. The analysis was performed by VeriSeq NIPT Solution v2 powered by Illumina NGS technology. Test menu options were expanded to include common aneuploidies (chromosomes 21, 18, and 13), all rare autosomal aneuploidies (RAAs), sex chromosome aneuploidies (SCAs), and partial deletions and duplications, referred to as copy number variation (CNVs), ≥7 Mb in size. The VeriSeq NIPT Solution v2 incorporates workflow, instrument, and software innovations performing clinical prenatal aneuploidy screening.

The workflow of the analysis Ided cIDNA isolation from maternal plasma, library preparation, next-generation sequencing (NGS), data analysis, interpretation, and reporting. DNA libraries will be sequenced by NextSeq550DX sequencer (Illumina, San Diego, CA, USA).

### 2.5. Statistical Analysis (Sample Size and Data Analysis)

This was a pilot study with no a priori hypotheses. A formal sample size calculation cannot thus be performed. All patients who met the inclusion criteria were included in the study. Descriptive statistics were performed. Data are presented as mean ± standard deviation (SD) and as median and interquartile range (IQR) for normally and not normally distributed continuous variables respectively, and as absolute frequency and relative percentage for categorical variables. Since pregnancy tissue genetic analysis is considered the gold standard for the product of conception chromosomal abnormalities assessment, we tested the diagnostic capacity of cfDNA by calculating: (i) sensitivity; (ii) specificity; (iii) positive likelihood ratio (PLR); (iv) negative likelihood ratio (NLR); (v) positive predictive value (PPV); (vi) negative predictive value (NPV); (vii) accuracy.

### 2.6. Data Collection

An electronic case report form (eCRF) was created for data collection. The study data were collected prospectively and examined through the Research Electronic Data Capture data collection system (RED Cap) of the A. Gemelli University Hospital Foundation, IRCCS (https://redcap-irccs.policlinicogemelli.it/, accessed on 1 December 2022). RED Cap is a secure system, created to collect research data by providing (1) a validated data collection system, (2) examination and transfer of the data, (3) automated data transfer procedures for statistical evaluation, and (4) import of data from external sources. Only researchers officially registered in the study or data managers were able to access the RED Cap platform through secure authentication and analyze the data.

## 3. Results

### 3.1. Patients

**Figure 1:**
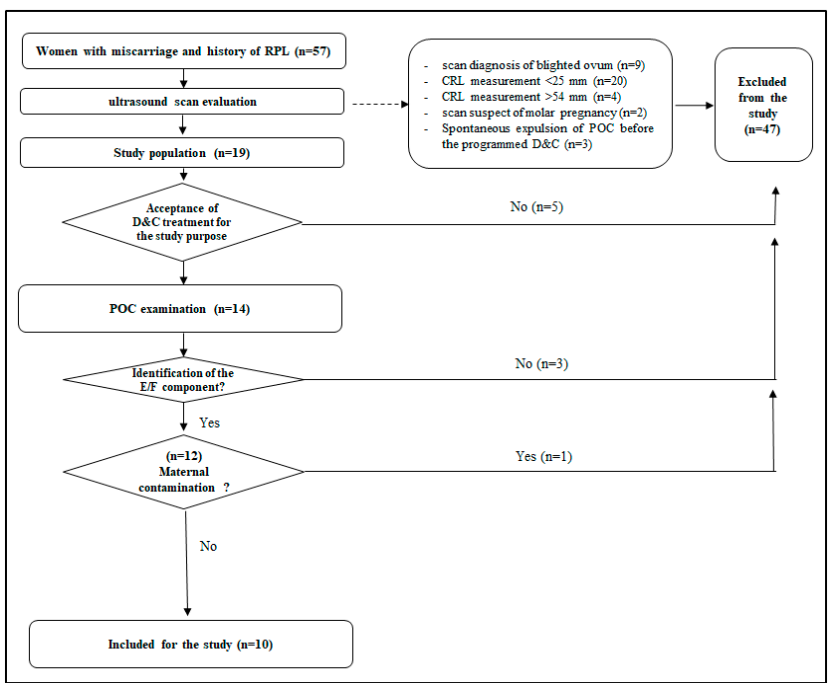
Study population. Fifty-seven women diagnosed with miscarriage and a history of RPL were considered for study entry. After ultrasound examination, a total of 38 women were excluded. A total of 5 women refused to participate to the study. The remaining 14 underwent D&C. After D&C, 4 cases were excluded. The final study population included 10 women.

Fifty-seven women diagnosed with miscarriage and a history of RPL were considered for study entry. After ultrasound examination, a total of 38 women were excluded. Of these, 9 were diagnosed with an anembryonic pregnancy loss (blighted ovum), 24 showed an inadequate CRL measurement (in 20 women the measurement of CRL was <24 mm and in 4 women >54 mm), 2 women had US criteria suggestive for molar pregnancy and 3 women had a spontaneous expulsion of the product of conception before the programmed D&C. A total of 5 women refused to participate to the study. The remaining 14 underwent D&C. After D&C, 4 cases were excluded because of the lack of an identifiable embryo-fetal component (*n* = 3) or maternal cell contamination (*n* = 1). The final study population included 10 women (Figure 1).

The mean age was 36.6 ± 3.4 years, the mean US gestational age at diagnosis was 10 weeks + 0 days and the amenorrhea was 12 weeks + 1 days long. The mean fetal fraction (FF) at cfDNA assessment was 9.1 + 4.2%. The clinical characteristics of included patients are shown in Table 1.

### 3.2. Chromosomal Microarray Analysis of the Product of Conception

CMA revealed a normal karyotype in 4/10 cases (40%), and chromosome aneuploidies in 6/10 (60%), consisting of trisomy 21 in 5 cases and monosomy X in the last case. In one of the cases showing a trisomy 21, a maternally inherited dup (9)(q21.32 q21.33) (size 0.6 Mb) was also detected.

### 3.3. Cell-Free DNA Analysis

CfDNA analysis gave normal results in 5/10 cases (50%). Results were abnormal in the remaining 5 cases (50%). All abnormal results were consistent with trisomy 21. One of the cases showing a trisomy 21 was associated with del(7)(p14.1p12.2) (size >10 Mb). Results are comparatively summarized in Table 2.

### 3.4. Comparison of the Diagnostic Yield of cfDNA Analysis and Chromosomal Microarray Testing of Miscarriage Tissues

When considering the diagnosis of trisomy 21 a full correspondence (100% of concordance) between cfDNA analysis and chromosomal microarray testing of miscarriage tissues was observed. By analyzing each individual result, cfDNA analysis failed to identify the only case of monosomy X in our cohort. Additionally, cfDNA analysis was not able to detect the maternally inherited dup (9)(q21.32 q21.33) in one case with trisomy 21, and it gave a false positive result, consisting of del(7)(p14.1p12.2) in another case of trisomy 21. The overall concordance is therefore 7/10 (70%). The cDNA diagnostic test properties are reported in Table 3.

## 4. Discussion

In the present pilot study, CMA and cfDNA results for fetal sex determination and fetal trisomy 21 assessment are superimposable. However, some discrepancies were also observed. To note, cfDNA analysis failed to identify the only case of monosomy X in our cohort. Furthermore, in one case with trisomy 21, cfDNA testing was not able to detect a maternally inherited dup(9)(q21.32 q21.33). The size of dup9 was 0.6 Mb, which is below the limit of cfDNA resolution. Finally, in a further case of trisomy 21, cfDNA testing reported a false positive result, consisting of del(7)(p14.1p12.2). Although both methods detected trisomy 21, meaning that the analysis was adequate in both cases, the cfDNA detected an additional result of del(7)(p14.1p12.2). Since the size of the deletion was calculated to be >10 Mb, we highly suggest that this discrepancy is due to placental mosaicism. Finally, a true fetal low-grade mosaicism (<20%) cannot be excluded due to the limit of CMA in detecting low levels mosaicism.

In a clinical perspective, the missing diagnosis of monosomy X is of particular relevance. In a recent systematic review and meta-analysis aimed at determining the accuracy of cfDNA for detecting chromosome aneuploidies (SCA) in singleton pregnancies, authors calculated, for 45 X, a sensitivity of 98.8% (95%CI 94.6–100%), a specificity of 99.4% (95%CI 98.7–99.9%) and a PPV of 14.5% (95%CI 7.0–43.8%) [35]. The size of our sample is obviously insufficient to draw conclusions. Considering the data reported by the meta-analysis mentioned above, this is an unexpected finding. The reasons behind this misdiagnosis are not known. However, it should be noted that the fetal fraction of this sample was one of the lowest in the study (4%, Table 2).

To date, few studies have tried to evaluate the possible role of cfDNA testing in pregnancy loss. An initial study demonstrated that cfDNA levels were higher in women who had an abortion compared with those who did not, thus suggesting that fetal demise might determine an increased release of apoptotic fetal cells [36]. Subsequently, Clark et al. investigated non-viable pregnancies at all gestational ages. By analyzing 38 cases, they found that, in pregnancies over 8 weeks of gestation, cfDNA was present in the maternal plasma with a fetal fraction greater than 3.7%, in about 76% of cases [37]. They thus proposed 8 weeks of gestation as cut-off, above which cfDNA analysis can provide adequate results. Some authors have subsequently questioned the cut-off proposed by Clark, pointing out that cfDNA analysis techniques based on genome-wide sequencing or on methods of fetal fraction enrichment can identify aneuploidies even on samples with a fetal fraction lower than 3.7% [37,38,39]. Two more recent studies tried to make a comparison between cfDNA analysis and POC karyotyping after pregnancy loss. Yaron et al. conducted the largest study in this research area. By using chorionic villous sampling, a collection technique not routinely used for POC sample collection, they analyzed 86 patients with EPL and then compared standard karyotyping of POCs and maternal plasma cfDNA analysis [40]. They distinguished the sensitivity of cfDNA according to both standard and pregnancy loss-specific likelihood ratios (LLR). When referring to the standard LLR thresholds used for noninvasive prenatal screening, the sensitivity of cfDNA in detecting aneuploidy was 55% with a specificity of 100%. When using pregnancy-loss-specific LLR thresholds, the sensitivity of cfDNA in detecting aneuploidy was 82% and the specificity was 90%, thus suggesting that cfDNA testing is an adequate alternative to the cytogenetic analysis of POCs in RPLs. More recently, Colley et al. analyzed samples from 57 patients with miscarriage at a gestational age <12 weeks [41]. The average cfDNA fetal fraction was 6% (2–19%). In total, 75% (43/57) of results were correctly identified by cfDNA. The authors stratified their results according to the FF and observed that, in the presence of an FF ≥9, 100% of cytogenetic results were correctly identified. The principal limitation of our study was the small sample size, which limits the reliability of the calculated diagnostic capacity of cfDNA. It could therefore be hypothesized that the calculated frequency of karyotype abnormalities could be attributed to chance. On the other hand, our results are in line with the data already reported in the literature and with what is expected on the basis of the age and clinical history of the included patients [42]. Our study should be considered as a pilot study conducted on a very selected population and that is in line with our previous efforts to improve the genetic information in couples with RPL [43]. Our results can be used as a rationale for validating cfDNA on a larger cohort of women diagnosed with pregnancy loss. Based on our data alone, no inferences regarding the clinical transferability of cfDNA in the management of abortion can be made. If, however, our data are combined with those of previous contributions, some reflections can be made. The cfDNA testing appears to be a valid alternative to the traditional methods to detect embryonic/fetal karyotype, when it is expected that the abortive tissue may be unavailable or insufficient to carry out the analysis. This situation frequently occurs in cases of spontaneous abortion treated pharmacologically (i.e., medical management). Collectively, available data also suggest an association between the fetal fraction value and the reliability of cfDNA testing. Prospective studies with an adequate sample size are indispensable before proposing the introduction of cfDNA testing into clinical practice to determine the karyotype of POC in women diagnosed with first trimester spontaneous abortion with pregnancy tissue still in situ.

Our efforts have the final aim of contributing to the better management of couples with RPL. In previous research we investigated the contribution of the “maternal side” to pregnancy loss. In particular we evaluated the role of autoimmune disorders, metabolic alterations, and endometrial inflammation/disfunction in the pathogenesis of pregnancy loss [44,45,46,47,48,49,50,51]. Further and larger studies are needed to better understand the contribution of the “fetal side” on the pregnancy loss.

## Figures and Tables

**Table 1:** Characteristics of patients and pregnancy in the study population.

Maternal age (years) + SD	36.6 ± 3.43
BMI (Kg/m^2^)	22.02 ± 2.03 (18–25)
Number of previous losses	4 (3–6)
Gestational age (days) + SD (weeks + days)	70.3+ 9.51 (10 + 0)
Amenorrhea age (days) + SD Amenorrhea age (weeks + days)	84.8 + 6.4 (12 + 1)
CRL (mm) + SD	33.34 + 13.9
Time from embryo demise (days + SD)	14.5 + 10

SD standard deviation; BMI body mass index; CRL crown rump length.

**Table 2:** Analysis of cfDNA using a modified Illumina VeriSeq non-invasive prenatal testing (NIPT) solution v2 workflow compared with the genetic outcomes of microarray analysis of the products of conception (POC).

Patients	BMI (kg/m^2^)	Age (Years)	GA Wks + Days (Days)	AA Wks + Days (Days)	CRL (mm)	CMA Results	CfDNA Results	Overall	FF (%)	CMA Sex (M/F)	CfDNA Sex (M/F)
1	23.15	36	10 + 2 (72)	12 + 1 (85)	34	Trisomy 21 and dup 9: arr[GRCh37] 9q21.32q21.32q21.33 (86774450_87406657) × 3 (size 0.6 Mb)	Trisomy 21	Partial	5%	F	F
2	21.26	37	12 + 0 (84)	12 + 2 (86)	54	Trisomy 21	Trisomy 21	Yes	12%	M	M
3	21.23	38	10 + 3 (73)	12 + 2 (86)	36	Trisomy 21	Trisomy 21	Yes	14%	F	F
4	20.70	34	9 + 1 (64)	10 + 1 (71)	24	no anomaly detected	no anomaly detected	Yes	3%	M	M
5	21.63	41	9 + 3 (66)	12 + 4 (88)	26	Trisomy 21	Trisomy 21	Yes	11%	F	F
6	22.04	33	12 + 0 (84)	13 + 1 (92)	54	no anomaly detected	no anomaly detected	Yes	12%	M	M
7	23.8	40	9 + 1 (64)	11 + 6 (83)	24	X monosomy	no anomaly detected	No	4%	F	F
8	17.6	31	9 + 1 (64)	11 + 6 (83)	24	no anomaly detected	no anomaly detected	Yes	14%	F	F
9	22.8	33	9 + 6 (69)	11 + 3 (80)	30	no anomaly detected	no anomaly detected	Yes	7%	M	M
10	24.7	39	9 + 6 (69)	13 + 3 (94)	30	Trisomy 21	Trisomy 21 and Del(7)(p14.1p12.2) (size > 10 Mb)	Partial	9%	M	M
Total	22.02 ± 2.03	36.6 ±3.4	10 + 0 (71 ± 7.24)	12 + 1 (84.8 ± 6.4)	34 ± 11				9.1 ± 4.2		

SD, standard deviation; BMI, body mass index; GA, gestational age; AA, amenorrhea age; Wks, weeks; CRL, crown rump length; CMA, chromosomal microarray analysis; cfDNA, cell-free DNA; FF, fetal fraction.

**Table 3:** Characteristics of cfDNA as diagnostic test for karyotype abnormalities of the miscarried tissue.

Statistic	Value	95% CI
Sensitivity	83%	36% to 100%
Specificity	100%	40% to 100%
Positive Likelihood Ratio	//	//
Negative Likelihood Ratio	0.17	0.03 to 1.00
Disease prevalence	60%	26% to 88%
Positive Predictive Value	100%	
Negative Predictive Value	80%	40% to 96%
Accuracy	90%	56% to 100%

## Data Availability

Due to privacy restrictions data are available after request to the first author.
